# Toxicological Evaluation of a Mixture of* Astragalus membranaceus* and* Panax notoginseng* Root Extracts (InnoSlim®)

**DOI:** 10.1155/2019/5723851

**Published:** 2019-07-01

**Authors:** Timothy S. Murbach, Róbert Glávits, John R. Endres, Gábor Hirka, Adél Vértesi, Erzsébet Béres, Ilona Pasics Szakonyiné

**Affiliations:** ^1^AIBMR Life Sciences, Inc., 2800 East Madison Street, Suite 202, Seattle, WA 98112, USA; ^2^Toxi-Coop Zrt., Magyar Jakobinusok Tere 4/B, H-1122 Budapest, Hungary

## Abstract

*Astragalus* spp. and* Panax* spp. have a long history of traditional human use. A blend, InnoSlim®, of highly purified and fractionated root extracts from* Astragalus membranaceus *and* Panax notoginseng *has now been developed for human consumption; however, the unique constituent content of this blend has not been specifically evaluated with respect to safety. Therefore, the toxicological potential of the blend was formally investigated in a series of studies—genetic toxicity was evaluated in a bacterial reverse mutation test followed by an in vivo mammalian micronucleus test, and general toxicity was evaluated in a 28-day repeated-dose oral toxicity study in rats. No evidence of mutagenicity was observed in the bacterial tester strains used, and no evidence of in vivo chromosomal damage resulting in increased frequency of micronucleated cells was observed in male Crl:NMRI BR mice. No mortality or toxic effects were observed, and no target organs were identified, in male and female Han:WIST rats exposed to 0, 400, 800, or 1200 mg/kg bw/day of the blend by gavage for 28 consecutive days. The highest dose—1200 mg/kg bw/day—was determined to be the NOAEL. Based on these results, extrapolation towards a safe human consumption level can be explored.

## 1. Introduction

Both* Astragalus membranaceus *and* Panax notoginseng* roots have long histories of traditional use.* A. membranaceus* Fisch. ex Bung, also known as huang qi (Chinese), membranous milkvetch root (English), ogi (Japanese), hwanggi (Korean), and astragel (Danish), has been in use for over 2000 years and, together with* A. membranaceus *var.* mongholicus*, is defined as* Radix Astragali* in the Pharmacopoeia of the People's Republic of China [1, 2]. It is an adaptogen that has been classified as a Qi and blood tonic in the Chinese Materia Medica and has been used in China for general weakness, to invigorate liver and spleen functions, to invigorate “yang”, and for overall vitality [3–6]. Traditionally, it has been mainly used as raw dried root, honey cured root, or as an aqueous decoction, although steeping in ethanol (or “spirits”) has also been described [1, 7]. Traditional intake has been reported to range from 9 to 30 g daily; however, use of greater than 60 g has been reported [1, 8].

The main constituents of* A. membranaceus *are saponins, flavonoids, polysaccharides, amino acids, and trace elements [9]. Saponins identified include 12 triterpene-oligoglycosides: astragalosides I-VIII, acetylastragaloside I, isoastragalosides I and II, and soyasaponin [10–12]. Flavonoids identified in* A. membranaceus* are calycosin-7-*O*-*β*-D-glucoside, calycosin-7-*O*-*β*-D-glucoside-6′-*O*-malonate, ononin, (6a*R*,11a*R*)-3-hydroxy-9,10-dimethoxypterocarpan-3-*O*-*β*-D-glucoside, calycosin, (3*R*)-7,2′-dihydroxy-3′,4′-dimethoxyisoflavan-7-*O*-*β*-D-glucoside, formononetin-7-*O*-*β*-D-glucoside-6′-*O*-malonate, and formononetin [13].

*P. notoginseng* (Burkill) is also known by the synonym* P. pseudoginseng* Wall. var.* notoginseng* (Burkill), basionym* Aralia quinquefolia* (L.) Decne. & Planch. var.* notoginseng* Burkill, and traditional Chinese names, Sanqi, San Qi, Shan Qi, Tu San Qi, Tianqi, and Tien–chi. The earliest description was written in the 16^th^ century by Li Shi Zhen in “*Bencao Gangmu* (Compendium of Materia Medica)” [14], and farmers have cultivated the root for over 1000 years because it was heavily depended upon for its reported benefits [14–20]. Typical total daily intake has been reported to range from approximately 3.12 to 15 g of a decoction or 1 to 5 g of ground powder (depending on interpretation of the Chinese measures qian and fen), and the use of ethanolic extractions has also been reported [14, 16, 19, 21].

The plant belongs to the* Panax* genus, which includes Asian ginseng and American ginseng (both of which, also, have long histories of human consumption), and analyses of 5S-rRNA from* Panax* species show a high degree of genetic similarity; approximately 75% of the ribosomal genome is conserved throughout the genus [15]. The main active constituents in* P. notoginseng* are saponins (56 have been identified through HPLC), which comprise approximately 8–13% by weight of the root. All of the saponins in notoginseng are classified as dammarane saponins; 21 are protopanaxtriols and 35 are protopanaxdiols. The first saponins isolated were the ginsenosides Rb1, Rd, Re, and Rg1 [14]. Other constituents include amino acids, the most prevalent being arginine, aspartic acid, and glutamic acid; five polyacetylenes: panaxytriol, panaxydol, notoginsenic acid, *β*-sophoroside, and 10-hydroxydeca-4,6-diynoic acid; the flavonoids quercetin and quercetin-3-*O-*sophorside; and the following phytosterols: *β*-sitosterol, daucosterol, betulin, lupeol, and calenduladiol [14, 15].

Modern research continues to build on traditional knowledge regarding uses of these botanicals, and a novel blend, InnoSlim®, of a hydroethanolic root extract of* A. membranaceus *and an aqueous root extract of* P. notoginseng *was developed to exploit properties of the botanicals related to metabolic regulation and weight management [22–25]. However, the safety of this unique preparation has not been formally explored although the long history of use, at substantial levels, of both of these botanicals provides an indication as to their safety. In addition, poorly characterized aqueous extracts of both* A. membranaceus *and* P. notoginseng *have been the subject of some toxicologic investigations, and both have produced generally negative results in genetic and oral toxicity tests [26–28] although an* Astragalus* root extract caused gastric erosion at the highest dose tested (described as 180 g/kg bw/day) [27]. A solvent-extracted* A. membranaceus* root was also investigated via parenteral administration and did not cause adverse effects in rats or dogs [29]. Nonetheless, while corroborative of safety, these studies were generally poorly described, and it is also unclear how similar or dissimilar the tested extracts are to the extracts that comprise InnoSlim® and, therefore, how relevant they are to the safety of this unique blend. Due to interest in InnoSlim® as a food ingredient, in the current work, we conducted a battery of toxicological investigations with the aim of understanding the potential toxic effects of the preparation as formulated.

## 2. Materials and Methods

### 2.1. Test Item

InnoSlim® is an equal mixture of dried extracts of* A. membranaceus* (10:1 hydroethanolic extract) and* P. notoginseng* (50:1 aqueous extract) roots blended together with a small amount of maltodextrin as an excipient and its production is compliant with current Good Manufacturing Practice. The final blend is a beige to light yellow powder with a characteristic odor and taste and is standardized to contain ≥2.5% saponins, ≥0.01% astragaloside I, and ≥1.0% ginsenoside Rg1. InnoSlim®, lot number C20171110, was provided by NuLiv Science USA, Inc., Brea, California, USA for use as the test item in the current work.

The test item meets food grade specifications for identity, physical characteristics, and contaminants, such as pesticide residues, organic solvents, heavy metals, and microbial growth. The manufacturing process uses an extraction and processing technology, under appropriate process controls, comprised of typical process steps including washing, pulverizing, extraction, chromatographic fractionation, concentration, drying, and blending. The raw botanicals are purchased from established growers and verified for identity, as well as assessed for moisture and ash content, heavy metals, and pesticide residues. The studies herein described were conducted in compliance with OECD Principles of Good Laboratory Practice (GLP) [30], except for the following deviation: analytical control of the test item formulations for homogeneity and stability was not performed because no appropriate test method was available. As previously described [31], animal husbandry was according to OECD test guidelines [32, 33] and the standard operating procedures (SOP) of the laboratory as well as relevant regulations for the protection, care, and use of animals.

### 2.2. Bacterial Reverse Mutation Test

The test was conducted according to OECD Guideline for Testing of Chemicals 471 [34] in order to evaluate the mutagenic potential of InnoSlim®. SOPs of the laboratory were applied as well as procedures described by Ames et al. [35], Maron and Ames [36], Kier et al. [37], Venitt and Parry [38], and Mortelmans and Zeiger [39].

In order to identify an appropriate vehicle and test concentrations for the main tests, a non-GLP preliminary solubility test and a concentration range finding test in* Salmonella typhimurium* TA98 and TA100 using a plate incorporation method were conducted. Based on the solubility test, ultrapure water (ASTM Type 1, prepared in the laboratory by Direct-Q5 system, Millipore) proved to be a suitable vehicle for the test item in a solution of top agar and phosphate buffer. Bacterial tester strains* Salmonella typhimurium* TA98, TA100, TA1535, and TA1537 and* Escherichia coli* WP2* uvrA* (Moltox, Inc., Boone, NC, USA) were utilized for the main tests with test item concentrations of 5000, 1600, 500, 160, 50, and 16 *μ*g/plate with and without metabolic activation (S9-mix). Standard, strain specific, verified positive controls, and appropriate positive control specific vehicles were utilized as previously described [40, 41]. The S9-mix was prepared in the laboratory with rat liver S9 fraction (Moltox, Inc., Boone, NC, USA) and was both certified by the supplier and verified in the laboratory for effectiveness with the positive controls.

The GLP main bacterial reverse mutation tests employed a standard plate incorporation procedure as the initial test and a preincubation procedure as the confirmatory test. Both tests were carried out in triplicate, and the test solutions, positive control solutions, and the S9-mix were freshly prepared at the beginning of each experiment. As described previously [40], the experimental data was collected and tabulated and results were evaluated on the basis of biological relevance according to the validated criteria developed by the laboratory.

### 2.3. In Vivo Mammalian Micronucleus Test

Bone marrow of specific pathogen-free (SPF) male Crl:NMRI BR mice (Toxi-Coop, Budapest, Hungary) was evaluated in order to investigate the potential of InnoSlim® to cause chromosomal damage in vivo. The study was conducted in accordance with OECD test guideline 474 [33], and procedures described by Salamone and Heddle [42] were also utilized.

Distilled water (Parma Product Kft., Budapest, Hungary) was selected as the negative control and vehicle for the test item, and dosing solutions were formulated by adding distilled water to the necessary mass of test item and stirring until homogeneity was reached. As no formulation analysis was performed, the dose formulations were freshly prepared each day and administered within two hours. The positive control was cyclophosphamide (Sigma-Aldrich Co., Saint Louis, MO, USA) dissolved in sterile water (NATURLAND Kft., Budapest, Hungary). Dose and sex selection for the main study were made on the basis of a non-GLP preliminary toxicity test conducted in male and female mice at the limit dose (2000 mg/kg bw).

Test item doses of 0, 500, 1000, and 2000 mg/kg bw were administered at a constant volume of 20 mL/kg bw by gavage to groups of five male mice with each animal receiving two consecutive doses 24 hours apart. An additional group of five mice was given the positive control once at a dose of 60 mg/kg bw intraperitoneally at a volume of 10 mL/kg bw. Following dosing and until sacrifice (24 hours after the final treatment), the mice were closely monitored for adverse reactions. A single bone marrow sample was obtained from the femurs of all animals immediately following sacrifice and prepared for microscopic examination to assess the proportion of polychromatic erythrocytes (PCE) among total erythrocytes and the frequency of micronucleated PCEs (MPCE).

### 2.4. 28-Day Repeated-Dose Oral Toxicity Studies in Rats

In general accordance with OECD test guideline 407 [43], the 28-day study was conducted in order to evaluate the toxic potential of InnoSlim® and determine a no-observed-adverse-effect level (NOAEL) in male and female SPF Han:WIST rats (Toxi-Coop, Budapest, Hungary).

Randomization by weight stratification was used to assign groups of 10 rats/sex to receive gavage (10mL/kg bw) administration of the test item at dose levels of 0, 400, 800, and 1200 mg/kg bw/day for 28 consecutive days. The vehicle and negative control were distilled water (Parma Product Kft., Budapest, Hungary). Due to the lack of stability data, the test item was carefully weighted and dissolved in the vehicle each day and administered within 4 hours of preparation.

All observations (except functional observations were not needed based on the results of the daily and weekly detailed clinical observations), measurements, and evaluations recommended in the cited test guideline were conducted. In addition, ophthalmologic examinations were conducted on all animals during the acclimation period and on control and high-dose animals at the end of the study, and body weight gain, feed efficiency, and organ weights relative to body and brain weights were calculated. All procedures were carried out according to the cited test guideline and/or laboratory SOPs. Mydriatic eye drops (Cicloplegicedol® (10 mg/mL), Laboratório Edol-Produtos Farmacêuticos S.A., Linda-a-Velha, Portugal) were administered prior to ophthalmoscopic examinations under subdued light, which was maintained in the animal room for the remainder of the examination days. Animals were fasted overnight after the final treatment, and, after weight measurement, Isofluran CP® anesthesia (Medicus Partner Kft, Biatorbágy, Hungary) was administered to induce narcosis. Sacrifice was by exsanguination from the abdominal aorta immediately following collection of blood samples from the retro-orbital venous plexus. Organ weights were determined, macroscopic examinations were conducted, and tissues and organs were preserved for histological examination.

### 2.5. Statistical Analyses

#### 2.5.1. In Vivo Mammalian Micronucleus Test

Kruskal-Wallis nonparametric one-way analysis of variance (ANOVA) was performed, using SPSS PC+ software, version 4 (SPSS, Inc., Chicago, IL, USA), to analyze MPCE frequencies. The data were checked for a linear trend in mutant frequency with treatment dose using the adequate regression analysis in Microsoft Excel version 2016 (Microsoft, Hungary). A* P*-value of <0.05 was considered statistically significant in all tests.

#### 2.5.2. 28-Day Repeated-Dose Oral Toxicity Studies in Rats

Heterogeneity of variance between groups was checked with Bartlett's homogeneity of variance test. If statistically significant heterogeneity was not detected, a one-way ANOVA was carried out, and positive results were further evaluated using Duncan's Multiple Range test to assess the significance of intergroup differences. Data was examined for normality using the Kolmogorov-Smirnov test if Bartlett's test was statistically significant, and non-normal distributions were further evaluated using Kruskal-Wallis nonparametric one-way ANOVA. Intergroup comparisons were performed post hoc using the Mann-Whitney U-test if nonparametric ANOVA results were statistically significant.

SPSS PC+ software, version 4, was used to conduct the above statistical analysis for the following data: body weight, body weight gain, food consumption, feed efficiency, clinical pathology, and absolute and relative organ weights. Male and female data were evaluated separately, and a* P*-value of <0.05 was considered statistically significant in all tests described. Statistical analysis of nonquantitative study parameter (clinical observations, ophthalmoscopy, and gross and histopathology) findings was not performed; frequencies of occurrence by sex and dose were calculated for these findings.

## 3. Results and Discussion

### 3.1. Bacterial Reverse Mutation Test

No precipitation of the test item was observed in any of the experiments nor was colony or background lawn development affected. No concentration-related or biologically relevant increases in revertant colony numbers of any of the five tester strains treated with the various test item concentrations with or without S9-mix were observed in the initial (Table 1) or confirmatory (Table 2) mutation tests. All observed variation remained within the corresponding historical negative control data ranges while the positive controls induced the expected positive responses within the data ranges of the corresponding historical positive controls.

### 3.2. In Vivo Mammalian Micronucleus Test

No mortality or adverse reactions to treatment were observed in any animals of the treatment or negative or positive control groups. MPCE frequencies for the negative and positive control groups were compatible with the historical control data of the laboratory, and a large, statistically significant increase in MPCE number was observed in the positive control compared to the concurrent and historical negative controls.

No statistically significant differences in the proportion of PCEs among total erythrocytes were observed in the 500, 1000, or 2000 mg/kg bw groups compared to controls although PCEs were slightly lower in the 2000 mg/kg group. No statistically significant increases in frequency of MPCEs were observed in the test item-treated groups compared to the concurrent negative controls, and the observed MPCE frequencies were compatible with the historical control data of the laboratory. Summary data for the micronucleus test is shown in Table 3.

### 3.3. 28-Day Repeated-Dose Oral Toxicity Study in Rats

#### 3.3.1. Clinical Observations and Ophthalmology

No mortality or morbidity occurred in any of the groups during the treatment period. With the exceptions of scars observed on the shoulders of a single male control animal from Days 17–22, no clinical signs or functional deficits were observed in any animals during the daily cage-side or weekly detailed clinical observations, and behavior and physical condition of the animals were normal during the entire observation period. On ophthalmologic examinations, the eyes of all animals examined appeared normal without any detected alterations.

#### 3.3.2. Body Weights and Food Consumption

**Figure 1:**
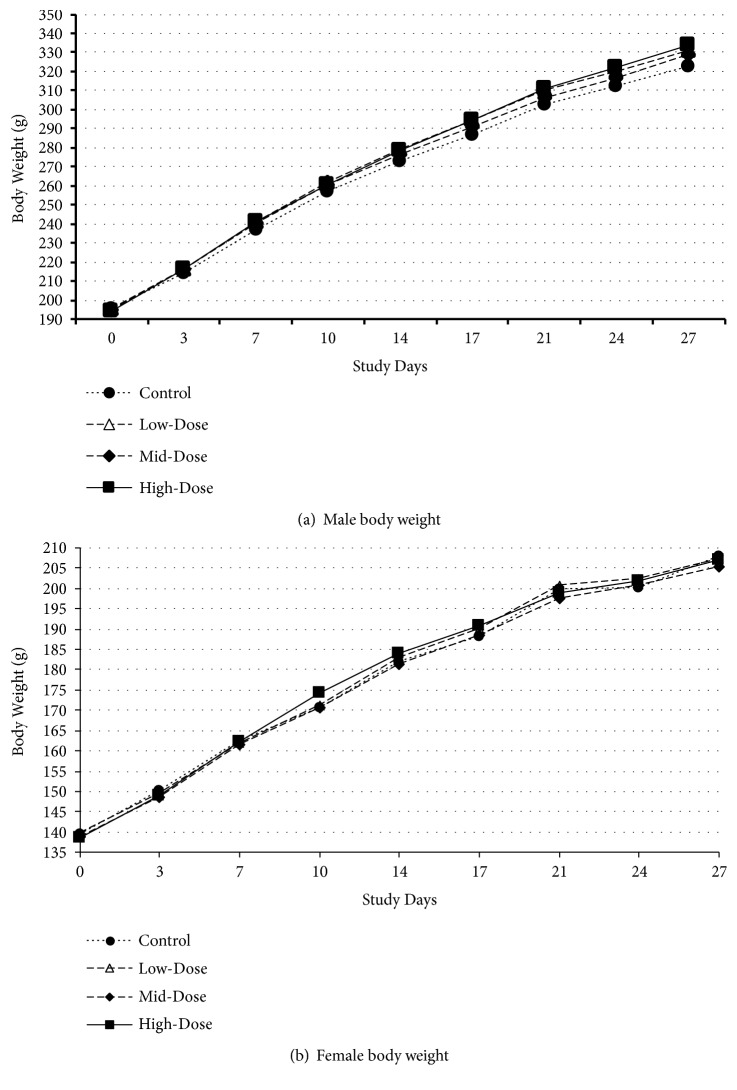
*Body Weights in the 28-Day Study*. Control = 0 mg/kg bw/day; low-dose = 400 mg/kg bw/day; mid-dose = 800 mg/kg bw/day; high-dose = 1200 mg/kg bw/day. (a) Male body weights. (b) Female body weights.

No statistically significant differences in mean body weights in treated groups compared to controls were observed in either sex (see Figure 1). A statistically significant increase in mean body weight gain was observed in the mid- and high-dose male groups between Days 0–3, and a statistically significant decrease in mean body weight gain was observed in the high-dose group of females between Days 17–21 (see Table S1 in the Supplementary Material). These transient changes, while dose-related, did not affect mean body weight or cumulative body weight gain and, therefore, were not considered test item-related.

Likewise, there were no statistically significant differences in food consumption compared to the respective controls in any groups of either sex while statistically significant differences in feed efficiency compared to controls were observed in mid- and high-dose males for Week 1 and mid-dose males for Week 4; no statistically significant differences in feed efficiency were observed in the female groups (see Table S2 in the Supplementary Material). The transient differences in feed efficiency observed in the male groups represent slight improvements with respect to controls but had no effect on body weight or body weight development.

#### 3.3.3. Clinical Pathology

Compared to the respective controls, analysis of hematology data detected a statistically significant increase in mean reticulocyte percentage in the high-dose male group and a statistically significant increase in mean hematocrit in the mid-dose female group. The following statistically significant changes compared to controls were detected on analysis of clinical chemistry data: decreased mean alanine aminotransferase activity in mid-dose males, decreased mean calcium and potassium concentrations and increased creatinine concentrations in low-dose females, and increased cholesterol in the high-dose females.

The above changes were not considered toxicologically relevant as they were of low magnitude (the alteration in calcium was marginally below the historical control range, and all others remained within their respective historical control ranges), appeared sporadic (although a dose-relationship could not be ruled out when the change occurred in the high-dose group (i.e., reticulocytes and cholesterol)), and were without correlating histopathology. The mean hematology and clinical chemistry data are presented in Tables 4 and 5.

#### 3.3.4. Gross Pathology

Pyelectasia was observed in the kidneys of 2 of 10 animals each in all male groups, 2 of 10 control and mid-dose females, and 1 of 10 low-dose females. In most cases, this was a single-sided finding with the exception of both high-dose males and one of the mid-dose females where both sides were affected. A diaphragmatic hernia was observed in a single male control animal, and point-like thymic hemorrhages were observed in a single female control animal. Slight to moderate hydrometra was observed in 2, 4, 2, and 2 female animals of the control, low-, mid-, and high-dose groups, respectively. No other gross lesions were observed in any animals. As the observed alterations occurred with similar incidence in controls and treated animals (or in controls only) without correlating histopathology (note some were associated with histological findings but were without inflammatory or other pathological lesions, see Section 3.3.6) and are common findings in untreated rats, they were considered incidental without toxicological relevance.

#### 3.3.5. Organ Weights

Statistically significant differences with respect to relevant controls were noted for slightly lower mean thymus weight (absolute and relative to body weight) in mid-dose male animals, slightly lower mean kidneys weight relative to body weight in mid- and high-dose female animals, and slightly lower mean heart weights (absolute and relative to body weight) in high-dose females (see Tables S3–S5 in the Supplementary Material). These differences, though statistically significant, were small in magnitude of change with respect to controls and remained within or were marginal to the historical control ranges (note thymus weights relative to body weights were below the historical control range in all male groups, including controls, and absolute heart weights of the female controls were above the historical control range). Additionally, there were no changes in related clinical pathology parameters or correlating histopathology. The weights of all other organs were similar in the control and test item treated groups. Therefore, the minor statistically significant variations in the organ weights were considered to have occurred sporadically without biological or toxicological significance.

#### 3.3.6. Histopathology

Renal pelvic dilatation without degenerative, inflammatory, or fibrotic changes or other related histopathological lesions was observed in correlation with the macroscopic observations. This also occurs in untreated rats [44–47] and its similar incidence in controls in the current work was considered indicative of an incidental finding without toxicological significance. Dilatation of the uterine horns also correlated with the macroscopic observations in control and high-dose females; however, microscopic examination was not extended to the low- and mid-dose group females as, due to its similar incidence in controls and lack of pathological lesions in related organs, this was considered a normal physiological process that occurs in the proestrus phase of the sexual cycle as a result of estrogen stimulation cycle [48, 49].

Mild acute thymic hemorrhage was observed microscopically in the same female control animal in which macroscopic hemorrhages were observed as well as in a single high-dose male animal. Mild acute hemorrhage was also observed in the lungs of a single high-dose male, and minimal alveolar emphysema was observed in one animal each of both the male and female control and high-dose groups. These three findings are also observed in untreated animals [47, 50, 51] and when taken together, in our experience, may be indicative of circulatory disturbances, dyspnea, and hypoxia that occur as a result of the exsanguination procedure. Due to their low incidence and similar occurrence in controls (with the exception of the pulmonary hemorrhage) in the current work, they were not considered toxicologically relevant.

The remaining microscopic findings were focal, subscapular interstitial fibrosis in the liver of a single control male, which was determined to be related to mechanical irritation of Glisson's capsule due to the diaphragmatic hernia observed macroscopically in the same animal, and minimal to mild hyperplasia of bronchus associated lymphoid tissue in a single animal each of the male control and high-dose groups and the female control group. Again, due to its mild degree without inflammatory changes, the low and similar incidence in the control and treated groups, and its occurrence in untreated animals [52, 53], the finding was considered incidental without toxicological significance. The histological findings are summarized in Table 6.

#### 3.3.7. Expanded Discussion

The mutagenicity of an aqueous extract of a* P. notoginseng* roots and rhizomes (described only as prepared following the “guidance of Traditional Chinese Medicine practice and Professor Wang Xingwen's “Proprietary Water Extraction Technology for Chinese Medicinal Plants””) was previously evaluated in* S. typhimurium* tester strains: TA97a, TA98, TA100, and TA102 using a plate incorporation procedure with and without metabolic activation [28]. No mutagenic activity was observed up to 5000 *μ*g/plate. These results are consistent with those observed in the current work conducted according to OECD protocols in tester strains* S. typhimurium* TA98, TA100, TA1535, and TA1537 and* E. coli* WP2* uvrA*, in which the test item (comprised of approximately 50%* P. notoginseng* root hydroethanolic extract) did not cause base pair substitution or frameshift mutations under the applied conditions up to 5000 *μ*g/plate.

The results of the current micronucleus test are also supported by previous works on both the aqueous* P. notoginseng* extract (PNS) of Jialing et al. described above and an aqueous extract of* A. membranaceus* root, which was described by Hui et al. as a “Huangqi* Astragalus membranaceus* composite,” (HAMC) in which the main ingredient was described as a water extract of the dried root of* A. membranaceus* [26]. While described as a composite, no other ingredients were identified by the authors although it was reported that dextran was used as a filler. Both studies were conducted in Kunming mice that were given PNS (up to 10 g/kg bw) or HAMC (up to 11 g/kg bw) twice at 24-hour intervals and euthanized six hours following the second treatment; bone marrow slides were evaluated for MPCEs by counting 1000 PCEs [26, 28]. No increases in MPCEs compared to the negative control were observed in either study. Additionally, HAMC and PNS did not exhibit genotoxic effects in sperm morphology tests in Kunming mice. In the current work, InnoSlim® did not induce chromosomal damage in the bone marrow of mice under the applied conditions of the OECD micronucleus test at doses up to 2000 mg/kg bw.

HAMC and PNS were also evaluated for acute and 30-day repeated-dose oral toxicity [26, 28]. In the acute studies, 22 g/kg bw HAMC or 20 g/kg bw PNS did not cause mortality and were well tolerated in Kunming mice observed for 14 days following dose administration. In the repeated dose studies in Wistar rats, no mortality or obvious toxic reactions were observed during the study periods, and no significant differences were observed in body weight, food intake and food utilization, hematology, blood chemistry, or organ weight and organ/body weight ratios at doses of HAMC up to 22 g/kg bw/day or PNS up to 1100 mg/kg bw/day, the highest doses tested. Additionally, HAMC did not cause adverse effects on gross and histopathological evaluations (while in the PNS study, Jialing et al. reported “organ pathological examination on liver, kidney, spleen, testis, ovary and gastrointestinal organs were performed,” no results were reported).

A decoction made from raw* Astragalus* root (species not identified) has also been evaluated in Wistar rats in a 90-day repeated-dose study [27]. The test item was prepared by boiling 900 g of raw dried* Astragalus* root. The decoction was strained, 1000 mL of solution set aside, and the root was boiled a second time to obtain another 1000 mL solution. The obtained solutions were then combined and boiled to reduce to a 1000 mL final solution, which was refrigerated and reboiled weekly. 20 mL of the prepared solution was used as the high-dose and was diluted with normal saline to produce the mid- and low-dose solutions. According to the authors, the low-, mid-, and high-doses were equivalent to 45, 90, and 180 g/kg bw/day of the dried* Astragalus* root. No death or abnormal clinical signs or statistically significant differences on body weight; food intake; hematological, clinical chemistry, or urinalysis parameters; or organ weights were observed in any groups, and no gross or histopathological lesions were observed in the low- and mid-dose animals. In the high-dose group, gastric ulcers and punctiform petechiae were observed macroscopically with microscopic evidence of associated inflammation in 8 of 10 animals.

The parenteral toxicity of* A. membranaceus* root has also been evaluated in rats and dogs [29]. A purified, freeze–dried, lyophilized powder containing polysaccharides and saponins (astragalosides I, II, and IV, isoastragalosides I, II, and IV, and acetylastragaloside I) was derived from an organic solvent extraction of powdered* A. membranaceus* root and administered intraperitoneally in Sprague-Dawley rats and intravenously in beagle dogs for 90 days. The organic solvent was not further identified. The tests were conducted according to standard protocols of guidelines for chronic toxicity testing of nature medicine and TCM, issued by State Food and Drug Administration of China, 2005, but further details were not provided. From limited results reporting it appears that there were no deaths and no adverse effects on body weight or clinical chemistry parameters and no test item-related histopathological findings at doses up to 39.9 g/kg bw/day in rats and 19.95 g/kg bw/day in dogs.

The previous acute and subchronic oral studies of aqueous extracts of* A. membranaceus* and* P. notoginseng* roots as well as the subchronic parenteral studies of an organic solvent extract of* A. membranaceus* root support the findings of the current work in which no toxic effects were observed when administering InnoSlim® to rats for 28 consecutive days at doses up to 1200 mg/kg bw/day. While gastric ulcers were observed in the high-dose group rats given the* Astragalus* root decoction for 30 days, the dose administered was much higher than that to which rats were exposed in the current work. According to the authors, the high-dose of the decoction was the equivalent of 180 g/kg bw of the dried* Astragalus* root while the dose of* A. membranaceus* root extract administered in the current work can be said to be equivalent to approximately 6 g/kg bw of raw* A. membranaceus* root. Additionally, the different constituencies extracted with water versus ethanol have not been characterized for comparison, and the species of* Astragalus* used to prepare the decoction is also unknown. For these reasons, ulcers observed following administration of the high-dose of the decoction do not detract from the results of the current work or present cause for concern with respect to consumption of InnoSlim®. Viewed cumulatively, the current work and previous works discussed above suggest that* A. membranaceus* and* P. notoginseng* roots are quite safe regardless of whether extracted with water or ethanol (and possibly other organic solvents).

## 4. Conclusions

The test item produced unequivocally negative results in the bacterial reverse mutation test and the in vivo mammalian micronucleus test. Therefore, under the applied conditions, InnoSlim® was not mutagenic up to the maximum recommended concentration for soluble noncytotoxic substances (5 mg/plate) and was not genotoxic in vivo when tested up to the limit dose (2000 mg/kg bw) in mice. Further InnoSlim® was not toxic when administered orally to rats at doses of 400, 800, and 1200 mg/kg bw/day for 28 consecutive days. The NOAEL was determined to be 1200 mg/kg bw/day (the highest dose tested) in male and female Han:WIST rats.

## Figures and Tables

**Table 1:** Summary of the results of the initial mutation test.

Initial Mutation Test (Plate Incorporation Test)																				
Concentrations (*μ*g/plate)	*Salmonella typhimurium* tester strains																*Escherichia coli*			
	TA 98				TA 100				TA 1535				TA 1537				WP2*uvrA*			
	-S9		+S9		-S9		+S9		-S9		+S9		-S9		+S9		-S9		+S9	
Mean values of revertants per plate Mutation rate (MR)	Mean	MR	Mean	MR	Mean	MR	Mean	MR	Mean	MR	Mean	MR	Mean	MR	Mean	MR	Mean	MR	Mean	MR
Untreated Control	15.7	1.09	15.3	0.82	62.3	0.88	77.7	0.77	8.3	0.89	9.7	1.21	7.7	0.92	6.0	1.06	34.3	0.75	48.3	1.06
*DMSO Control *	*13.7*	*1.00*	*16.0*	*1.00*	*–*	*–*	*92.0*	*1.00*	*–*	*–*	*9.7*	*1.00*	*6.0*	*1.00*	*7.0*	*1.00*	*–*	*–*	*44.0*	*1.00*
Ultrapure Water Control	14.3	1.00	18.7	1.00	70.7	1.00	100.7	1.00	9.3	1.00	8.0	1.00	8.3	1.00	5.7	1.00	46.0	1.00	45.7	1.00

TEST ITEM																				

5000	16.3	1.14	24.3	1.30	73.7	1.04	89.7	0.89	9.3	1.00	8.0	1.00	9.0	1.08	7.3	1.29	37.7	0.82	44.3	0.97
1600	14.0	0.98	19.0	1.02	68.3	0.97	96.7	0.96	10.0	1.07	9.0	1.13	7.0	0.84	4.0	0.71	42.0	0.91	54.3	1.19
500	13.7	0.95	23.3	1.25	85.3	1.21	108.0	1.07	10.3	1.11	10.7	1.33	9.0	1.08	4.7	0.82	48.7	1.06	47.3	1.04
160	16.7	1.16	18.7	1.00	79.3	1.12	113.7	1.13	11.0	1.18	11.0	1.38	5.7	0.68	5.0	0.88	44.7	0.97	51.0	1.12
50	11.3	0.79	17.0	0.91	68.7	0.97	101.3	1.01	10.3	1.11	11.7	1.46	7.3	0.88	11.3	2.00	40.0	0.87	44.3	0.97
16	19.7	1.37	25.0	1.34	72.7	1.03	101.7	1.01	10.7	1.14	11.0	1.38	7.0	0.84	6.7	1.18	35.7	0.78	55.0	1.20

POSITIVE CONTROLS																				

*NPD (4 μg/plate)*	*387.3*	*28.34*	*–*	*–*	*–*	*–*	*–*	*–*	*–*	*–*	*–*	*–*	*–*	*–*	*–*	*–*	*–*	*–*	*–*	*–*
*SAZ (2 μg/plate)*	*–*	*–*	*–*	*–*	*848.0*	*12.00*	*–*	*–*	*853.3*	*91.43*	*–*	*–*	*–*	*–*	*–*	*–*	*–*	*–*	*–*	*–*
*9AA (50 μg/plate)*	*–*	*–*	*–*	*–*	*–*	*–*	*–*	*–*	*–*	*–*	*–*	*–*	*245.3*	*40.89*	*–*	*–*	*–*	*–*	*–*	*–*
*MMS (2 μL/plate) *	*–*	*–*	*–*	*–*	*–*	*–*	*–*	*–*	*–*	*–*	*–*	*–*	*–*	*–*	*–*	*–*	*1053.3*	*22.90*	*–*	*–*
*2AA (2 μg/plate)*	*–*	*–*	*1757.3*	*109.83*	*–*	*–*	*2077.3*	*22.58*	*–*	*–*	*171.3*	*17.72*	*–*	*–*	*130.7*	*18.67*	*–*	*–*	*–*	*–*
*2AA (50 μg/plate)*	*–*	*–*	*–*	*–*	*–*	*–*	*–*	*–*	*–*	*–*	*–*	*–*	*–*	*–*	*–*	*–*	*–*	*–*	*241.0*	*5.48*

*MR*, mutation rate; *DMSO*, dimethyl sulfoxide; NPD, 4-Nitro-1,2-phenylenediamine; *SAZ*, sodium azide; *9AA*, 9-aminoacridine; *MMS*, methyl methanesulfonate; *2AA*, 2-aminoanthracene.

*Remarks*. Ultrapure water was applied as vehicle of the test item and the positive control substances: SAZ and MMS; and the DMSO was applied as vehicle for positive control substances: NPD, 9AA, and 2AA. The mutation rate of the test item, SAZ, MMS, and untreated control refers to the ultrapure water; the mutation rate of NPD, 9AA, and 2AA refers to DMSO.

**Table 2:** Summary of the results of the confirmatory mutation test.

Confirmatory Mutation Test (Pre-Incubation Test)																				
Concentrations (*μ*g/plate)	*Salmonella typhimurium* tester strains																*Escherichia coli*			
	TA 98				TA 100				TA 1535				TA 1537				WP2*uvrA*			
	-S9		+S9		-S9		+S9		-S9		+S9		-S9		+S9		-S9		+S9	
Mean values of revertants per plate Mutation rate (MR)	Mean	MR	Mean	MR	Mean	MR	Mean	MR	Mean	MR	Mean	MR	Mean	MR	Mean	MR	Mean	MR	Mean	MR
Untreated Control	12.7	0.97	17.7	0.70	69.3	1.10	91.0	0.93	11.0	0.79	11.3	0.87	6.7	0.83	8.7	0.93	26.7	1.01	37.3	1.24
*DMSO Control*	*14.7*	*1.00*	*25.3*	*1.00*	*–*	*–*	*85.3*	*1.00*	*–*	*–*	*12.0*	*1.00*	*6.0*	*1.00*	*9.3*	*1.00*	*–*	*–*	*35.3*	*1.00*
Ultrapure Water Control	13.0	1.00	25.3	1.00	63.0	1.00	97.7	1.00	14.0	1.00	13.0	1.00	8.0	1.00	9.3	1.00	26.3	1.00	30.0	1.00

TEST ITEM																				

5000	17.3	1.33	24.7	0.97	83.0	1.32	96.0	0.98	17.7	1.26	12.7	0.97	8.3	1.04	7.7	0.82	36.0	1.37	36.7	1.22
1600	15.3	1.18	23.3	0.92	78.0	1.24	98.7	1.01	12.7	0.90	12.3	0.95	8.7	1.08	9.0	0.96	28.3	1.08	35.7	1.19
500	18.0	1.38	20.3	0.80	71.3	1.13	94.3	0.97	13.0	0.93	13.0	1.00	6.3	0.79	9.7	1.04	39.7	1.51	37.3	1.24
160	12.0	0.92	23.7	0.93	85.3	1.35	104.3	1.07	12.0	0.86	10.0	0.77	9.3	1.17	9.3	1.00	29.7	1.13	40.0	1.33
50	11.0	0.85	19.0	0.75	76.7	1.22	99.3	1.02	11.3	0.81	13.7	1.05	6.7	0.83	8.0	0.86	31.7	1.20	31.0	1.03
16	17.0	1.31	21.3	0.84	69.3	1.10	104.3	1.07	11.0	0.79	13.3	1.03	8.7	1.08	9.3	1.00	30.3	1.15	40.0	1.33

POSITIVE CONTROLS																				

*NPD (4 μg/plate)*	*460.7*	*31.41*		*–*	*–*	*–*	*–*	*–*	*–*	*–*	*–*	*–*	*–*	*–*	*–*	*–*	*–*	*–*	*–*	*–*
*SAZ (2 μg/plate)*	*–*	*–*	*–*	*–*	*625.3*	*9.93*	*–*	*–*	*1157.3*	*82.67*	*–*	*–*	*–*	*–*	*–*	*–*	*–*	*–*	*–*	*–*
*9AA (50 μg/plate)*	*–*	*–*	*–*	*–*	*–*	*–*	*–*	*–*	*–*	*–*	*–*	*–*	*364.7*	*60.78*	*–*	*–*	*–*	*–*	*–*	*–*
*MMS (2 μL/plate) *	*–*	*–*	*–*	*–*	*–*	*–*	*–*	*–*	*–*	*–*	*–*	*–*	*–*	*–*	*–*	*–*	*1258.7*	*47.80*	*–*	*–*
*2AA (2 μg/plate)*	*–*	*–*	*677.3*	*26.74*	*–*	*–*	*726.7*	*8.52*	*–*	*–*	*107.0*	*8.92*	*–*	*–*	*145.7*	*15.61*	*–*	*–*	*–*	*–*
*2AA (50 μg/plate)*	*–*	*–*	*–*	*–*	*–*	*–*	*–*	*–*	*–*	*–*	*–*	*–*	*–*	*–*	*–*	*–*	*–*	*–*	*156.3*	*4.42*

*MR*, mutation rate; *DMSO*, dimethyl sulfoxide; *NPD*, 4-nitro-1,2-phenylenediamine; *SAZ*, sodium azide; *9AA*, 9-aminoacridine; *MMS*, methyl methanesulfonate; *2AA*, 2-aminoanthracene

*Remarks*. Ultrapure water was applied as vehicle of the test item and the positive control substances: SAZ and MMS; and the DMSO was applied as vehicle for positive control substances: NPD, 9AA, and 2AA. The mutation rate of the test item, SAZ, MMS, and untreated control refers to the ultrapure water; the mutation rate of NPD, 9AA, and 2AA refers to DMSO.

**Table 3:** Summary results of the in vivo mammalian micronucleus test.

*Groups (n = 5*†)	*Sampling time*‡	*Total PCEs analyzed*	*MPCE*		*PCE/(PCE+NCE)*	
			*(per 4000 PCE)*			
			mean	±SD	mean	±SD
Hist. Neg. Control	24	280000	5.11	0.98	–	–
Con. Neg. Control	24	20000	5.40	1.14	0.53	0.01
500 mg/kg bw	24	20000	5.00	1.41	0.54	0.02
1000 mg/kg bw	24	20000	5.20	0.84	0.52	0.01
2000 mg/kg bw	24	20000	5.40	0.89	0.48	0.01
Positive Control	24	20000	148.20*∗∗*	8.17	0.39	0.02

Con. Neg. Control, concurrent negative control; Hist. Neg. Control, historical negative control; MPCE, micronucleated polychromatic erythrocytes; NCE, normochromatic erythrocyte; PCE, polychromatic erythrocyte.

Positive Control: Cyclophosphamide (60 mg/kg bw).

†Historical negative control (n = 70).

‡Hours following last treatment.

*∗∗*p < 0.01 to the concurrent and historical negative control; Kruskall Wallis nonparametric ANOVA.

**Table 4:** Hematology in the 28-day study.

Group		WBC	NEU	LYM	MONO	EOS	BASO	RBC	HGB	HCT	MCV	MCH	MCHC	PLT	RET	PT	APTT
(mg/kg bw/day)		[x10^9^/L]	[%]	[%]	[%]	[%]	[%]	[x10^12^/L]	[g/L]	[L/L]	[fL]	[pg]	[g/L]	[x10^9^/L]	[%]	[sec]	[sec]
*Male*																	
0 (Control)	Mean	8.39	14.88	81.35	2.05	1.01	0.12	8.78	160.5	0.48	55.16	18.26	331.0	884.1	2.00	10.39	13.31
(n = 10)	SD	2.40	7.24	7.85	0.70	0.63	0.04	0.28	5.74	0.02	0.92	0.47	7.3	195.1	0.31	0.21	1.69
400	Mean	8.81	12.42	84.05	1.97	0.83	0.10	8.64	158.2	0.48	55.82	18.32	328.3	889.7	2.13	10.44	14.39
(n = 10)	SD	1.19	4.76	5.41	0.71	0.35	0.05	0.12	2.74	0.01	1.10	0.45	4.8	114.7	0.32	0.19	0.50
800	Mean	8.76	12.17	84.38	1.82	0.94	0.10	8.69	159.0	0.48	55.55	18.33	329.8	885.0	2.15	10.28	13.01
(n = 10)	SD	1.39	4.33	4.67	0.48	0.75	0.00	0.26	4.14	0.01	1.55	0.38	8.2	153.2	0.31	0.19	0.60
1200	Mean	8.62	12.86	83.77	2.04	0.74	0.10	8.60	156.2	0.48	55.64	18.19	327.0	856.3	2.52	10.27	12.94
(n = 10)	SD	1.72	3.37	4.00	0.58	0.27	0.05	0.45	9.17	0.03	1.47	0.67	13.8	64.6	0.35	0.25	1.25
	SS														*∗∗*		
Test for Significance		NS	NS	NS	NS	NS	NS	NS	NS	NS	NS	NS	NS	NS	DN	NS	NS
Historical Control Range		4.9–11.2	7.6–16.2	78.8–89.0	1.3–3.3	0.4-7.9	0.0–0.2	7.80–8.76	151–172	0.45–0.50	54.4–59.8	18.1–20.8	334–356	638–1100	2.37–4.04	9.8–10.7	9.9–15.8

*Female*																	
0 (Control)	Mean	7.19	15.57	80.77	1.92	1.00	0.10	8.45	154.9	0.47	56.17	18.36	327.2	866.8	2.46	10.05	13.63
(n = 10)	SD	1.45	3.81	4.08	0.44	0.24	0.05	0.42	4.1	0.01	1.72	0.59	5.7	134.8	0.36	0.27	1.25
400	Mean	5.77	15.23	80.98	1.91	1.28	0.09	8.49	156.2	0.48	55.94	18.43	329.4	912.0	2.56	10.14	14.03
(n = 10)	SD	0.85	4.67	5.01	0.55	0.41	0.03	0.32	6.4	0.01	1.11	0.39	6.4	92.9	0.34	0.25	1.44
800	Mean	6.62	11.41	84.90	1.66	1.35	0.09	8.54	157.5	0.49	57.19	18.44	322.4	893.3	2.40	10.03	14.07
(n = 10)	SD	2.04	2.10	2.90	0.47	0.97	0.06	0.26	4.5	0.01	1.63	0.45	5.7	122.0	0.48	0.29	1.10
	SS									*∗*							
1200	Mean	6.20	14.93	80.27	2.12	1.92	0.08	8.41	156.3	0.47	56.51	18.57	328.8	910.7	2.46	9.97	13.61
(n = 10)	SD	1.43	5.53	7.53	0.62	2.44	0.04	0.27	5.4	0.02	1.11	0.44	5.3	182.5	0.38	0.17	1.25
Test for Significance		NS	NS	NS	NS	NS	NS	NS	NS	DN	NS	NS	NS	NS	NS	NS	NS
Historical Control Range		3.5–8.3	6.5–26.9	67.7–90.8	0.9–3.2	0.6–4.4	0.0–0.2	7.47–8.85	145–172	0.41–0.49	52.6–60.0	17.8–20.8	327–361	768–1230	1.86–3.44	9.5–10.0	9.6–19.1

APTT, activated partial thromboplastin time; BASO, basophil granulocytes; DN, Duncan's multiple range test; EOS, eosinophil granulocytes; HCT, hematocrit; HGB, hemoglobin; LYM, lymphocyte; MCV, mean corpuscular volume; MCH, mean corpuscular hemoglobin; MCHC, mean corpuscular hemoglobin concentration; MONO, monocyte; NEU, neutrophil granulocytes; NS, not significant; PLT, platelet count; PT, prothrombin time; RBC, red blood cell (erythrocyte); RET, reticulocyte; SD, standard deviation; SS, statistically significant compared to control; WBC, white blood cell.

*∗*p < 0.05; *∗∗*p < 0.01.

**Table 5:** Clinical chemistry in the 28-day study.

*Group*		*ALT*	*AST*	*ALP*	*TBIL*	*CREA*	*UREA*	*GLUC*	*CHOL*	*Pi*	*Ca* ^*++*^	*Na* ^*+*^	*K* ^*+*^	*Cl* ^*-*^	*ALB*	*TPROT*	*A/G*
*(mg/kg bw/day)*		[U/L]	[U/L]	[U/L]	[*µ*mol/L]	[*µ*mol/L]	[mmol/L]	[mmol/L]	[mmol/L]	[mmol/L]	[mmol/L]	[mmol/L]	[mmol/L]	[mmol/L]	[g/L]	[g/L]	
*Male*																	
Control	Mean	55.00	91.60	165.20	1.26	24.70	8.28	6.50	2.40	2.54	2.78	145.06	4.66	98.57	44.01	61.96	2.5
(n = 10)	SD	6.73	13.65	22.29	0.36	3.16	0.65	0.50	0.57	0.26	0.10	1.11	0.28	1.11	1.80	3.22	0.2
400	Mean	50.30	86.70	175.50	1.27	24.30	7.96	6.71	2.24	2.42	2.73	144.95	4.42	98.95	44.12	60.95	2.6
(n = 10)	SD	9.74	10.36	26.71	0.44	1.89	0.81	0.41	0.23	0.16	0.06	1.17	0.21	1.18	1.11	1.98	0.1
800	Mean	44.80	81.50	179.90	1.34	22.70	7.51	6.17	2.45	2.71	2.79	144.66	4.53	97.78	44.40	62.29	2.5
(n = 10)	SD	5.39	7.71	24.28	0.45	2.21	0.96	0.49	0.40	0.16	0.06	1.45	0.12	0.94	1.09	2.17	0.2
	SS	*∗*															
1200	Mean	50.80	96.50	189.50	1.04	25.90	7.83	6.38	2.24	2.55	2.74	144.87	4.50	98.13	43.95	62.64	2.4
(n = 10)	SD	11.24	25.59	28.23	0.29	2.69	1.42	0.70	0.35	0.20	0.05	0.83	0.20	1.20	1.76	2.48	0.3
Test for Significance		DN	NS	NS	NS	NS	NS	NS	NS	NS	NS	NS	NS	NS	NS	NS	NS
Historical Control Range		35–61	72–136	123–262	0.8–2.3	17–29	5.6–10.9	4.0–5.9	1.7–3.0	2.5–3.3	2.6–3.0	141.4–147.6	4.3–5.6	94.5–101.8	39.9–45.8	55.5–63.4	2.0–3.1

*Female*																	
0 (Control)	Mean	43.80	88.80	115.40	1.37	24.80	6.91	6.32	1.70	2.28	2.72	143.17	4.38	99.61	46.00	62.05	2.90
(n = 10)	SD	9.48	13.64	22.57	0.63	1.23	0.71	0.36	0.23	0.27	0.05	1.56	0.39	1.69	1.52	1.55	0.31
400	Mean	40.90	81.80	102.30	1.42	27.50	7.00	6.18	1.62	2.04	2.64	142.03	4.01	98.47	46.56	63.35	2.81
(n = 10)	SD	3.93	8.93	17.59	0.32	1.78	0.93	0.55	0.33	0.24	0.11	1.90	0.33	1.48	1.48	3.17	0.34
	SS					*∗∗*					*∗*		*∗*				
800	Mean	47.90	91.40	102.10	1.41	24.40	7.20	6.48	1.99	2.15	2.72	143.85	4.29	100.37	48.27	63.65	3.19
(n = 10)	SD	9.62	17.23	19.68	0.40	1.71	1.49	0.86	0.46	0.29	0.07	1.79	0.25	2.03	2.98	2.91	0.44
1200	Mean	42.00	85.50	102.50	1.34	25.30	7.39	6.73	2.09	2.11	2.68	142.59	4.34	99.40	47.11	64.01	2.81
(n = 10)	SD	3.33	17.06	23.46	0.30	2.58	1.42	0.80	0.24	0.39	0.08	1.20	0.30	1.77	1.88	2.56	0.30
	SS								*∗*								
Test for Significance		NS	NS	NS	NS	DN	NS	NS	DN	NS	DN	NS	DN	NS	NS	NS	NS
Historical Control Range		26–60	67–145	66–188	0.9–2.0	21.0–32.0	4.8–12.2	3.3–6.2	1.4–2.5	1.8–2.8	2.5–2.7	139.8–147.3	3.7–4.7	97–103.7	42.0–50.3	58.7–68.4	2.2–3.8

A/G, albumin to globulin ratio; ALB, albumin; ALP, alkaline phosphatase; ALT, alanine aminotransferase; AST, aspartate aminotransferase; BAC, bile acids; Ca++, calcium; CHOL, cholesterol; Cl-, chloride; CREA, creatinine; DN, Duncan's multiple range test; GLUC, glucose; K+, potassium; Na+, sodium; NS, not significant; Pi, inorganic phosphorous; SD, standard deviation; SS, statistically significant compared to control; TBIL, total bilirubin; TPROT, total protein.

*∗*p < 0.05; *∗∗*p < 0.01.

**Table 6:** Summary of histopathology findings.

	Dose group (mg/kg bw/day)	Control	400	800	1200
Organs	Observations	n=10	N/A	N/A	n=10
*Male*					
	Animals with no microscopic findings	7/10	N/A	N/A	6/10
Kidneys:	Pelvic dilatation, slight	2/10^a,b^	2/2	2/2	2/10
Liver:	Focal interstitial fibrosis, mild	1/10^a^	/	/	0/10
Lungs:	Acute pulmonary hemorrhage, mild	0/10	/	/	1/10^c^
	Alveolar emphysema, minimal	1/10	/	/	1/10^d^
	Hyperplasia of BALT, minimal to mild	1/10^b^	/	/	1/10^d^
Thymus:	Acute hemorrhage, mild	0/10	/	/	1/10^c^

*Female*					
	Animals with no microscopic findings	5/10	N/A	N/A	7/10
Kidneys:	Pelvic dilatation, slight	2/10^e^	1/1	2/2	0/10
Lungs:	Alveolar emphysema, minimal	1/10	/	/	1/10
	Hyperplasia of BALT, minimal	1/10	/	/	0/10
Thymus:	Acute hemorrhage, mild	1/10^e^	/	/	0/10
Uterus:	Dilatation	2/10^e^	/	/	2/10

/, not examined; BALT, bronchus associated lymphoid tissue; N/A, not applicable (only animals with gross lesions were examined).

Data represent incidence of the observation (number of animals with observation per number of animals observed).

Organs without lesions in 10/10 control or high-dose animals not shown.

Matching superscripts represent findings observed in the same animal.

## Data Availability

The mean data sets generated and utilized for statistical analysis to support the findings of these studies are included within the article or in the supplementary information files. All other raw and processed data used to support the findings of these studies are available from the corresponding author upon request.

## Supplementary Materials

Supplementary Materials In order that all mean data sets subject to statistical analysis and upon which the authors drew interpretations and conclusions are available to readers, tables for body weight gain, food consumption, feed efficiency, and absolute and relative organ weights were provided as supplementary materials.
