# Supplementary material for: Toxicological Evaluation of a Mixture of Astragalus membranaceus and Panax notoginseng Root Extracts (InnoSlim®)
Source: J Toxicol. 2019 Jul 1;2019:5723851. doi: 10.1155/2019/5723851 (PMC6633876; doi:10.1155/2019/5723851)
Supplement: Supplementary Materials — In order that all mean data sets subject to statistical analysis and upon which the authors drew interpretations and conclusions are available to readers, tables for body weight gain, food consumption, feed efficiency, and absolute and relative organ weights were provided as supplementary materials. [file 5723851.f1.pdf]

Table S1. Body Weight Gain in the 28-Day Study

Group (mg/kg bw/day)		Body weight gain (g) between days 0-3	3-7	7-10	10-14	14-17	17-21	21-24	24-27	0-27
**Male**										
0 (Control)	Mean	18.5	22.7	19.9	16.0	13.9	15.9	9.5	10.5	126.9
(n = 10)	SD	2.0	3.2	3.2	3.7	2.4	3.2	1.8	3.1	11.8
400	Mean	20.4	25.3	20.8	17.3	14.7	15.9	9.7	11.4	135.5
(n = 10)	SD	2.3	4.0	3.4	3.5	3.1	2.5	3.1	3.3	10.9
	SS									
800	Mean	21.5	24.2	20.1	16.1	14.5	14.7	10.5	12.7	134.3
(n = 10)	SD	3.4	3.6	3.8	3.4	2.3	2.8	2.5	3.4	14.5
	SS	*								
1200	Mean	22.4	25.1	19.2	17.8	16.1	16.6	11.2	11.7	140.1
(n = 10)	SD	2.3	3.1	3.2	3.4	3.9	4.0	3.6	2.5	15.6
	SS	**								
Test for Significance		DN	NS	NS	NS	NS	NS	NS	NS	NS
**Female**										
0 (Control)	Mean	10.8	12.2	8.1	11.4	6.2	11.7	0.2	7.8	68.4
(n = 10)	SD	3.2	2.3	4.2	3.0	4.3	3.7	3.6	4.8	9.9
400	Mean	9.8	12.3	9.5	11.5	7.3	10.5	1.8	4.8	67.5
(n = 10)	SD	2.3	2.3	4.2	3.4	4.0	2.5	3.3	4.1	6.5
	SS									
800	Mean	9.8	13.0	8.8	10.7	7.4	9.1	3.2	4.3	66.3
(n = 10)	SD	2.1	2.9	4.9	3.9	5.1	3.0	3.4	3.7	10.6
	SS									
1200	Mean	10.5	13.1	12.1	9.8	6.8	8.1	2.8	5.2	68.4
(n = 10)	SD	2.6	3.7	4.7	4.3	3.0	3.5	2.7	4.8	14.5
	SS						*			
Test for Significance		NS	NS	NS	NS	NS	DN	NS	NS	NS

Abbreviations: DN, Duncan's multiple range test; NS, Not Significant; SD, standard deviation; SS, statistically significant compared to control.
*p < 0.05; **p < 0.01

Table S2. Food Consumption and Feed Efficiency in the 28-Day Study

Group (mg/kg bw/day)		Food consumption (g/animal/day)				Feed efficiency (g food/ g bwg)				
	Days	0-7	7-14	14-21	21-27	0-7	7-14	14-21	21-27	0-27
	Weeks	1	2	3	4	1	2	3	4	1-4
**Male**										
0 (Control)	Mean	22.3	23.7	24.1	23.8	3.81	4.68	5.72	7.30	5.01
(n = 10)	SD	1.6	1.9	1.9	1.9	0.28	0.58	0.55	1.21	0.26
400	Mean	23.3	24.4	25.6	24.0	3.59	4.51	5.92	7.01	4.86
(n = 10)	SD	1.5	2.0	1.8	2.0	0.28	0.46	0.77	1.22	0.34
800	Mean	22.5	23.1	23.9	23.1	3.47	4.53	5.76	6.07	4.67
(n = 10)	SD	2.0	1.6	1.7	1.5	0.30	0.47	0.62	0.79	0.26
	SS					*			*	
1200	Mean	22.8	24.0	25.5	24.4	3.37	4.58	5.58	6.55	4.68
(n = 10)	SD	2.4	2.4	2.9	2.7	0.39	0.54	0.96	1.09	0.49
	SS					**				
Test for Significance		NS	NS	NS	NS	DN	NS	NS	DN	NS
**Female**										
0 (Control)	Mean	16.0	17.3	18.3	17.7	5.03	6.69	7.63	16.88	6.93
(n = 10†)	SD	0.8	0.9	1.2	1.6	0.83	2.17	2.15	12.89	0.73
400	Mean	16.4	17.1	18.5	17.6	5.24	5.86	7.86	17.92	7.01
(n = 10†)	SD	1.7	1.5	1.5	1.3	0.62	0.99	2.50	13.67	0.88
800	Mean	16.4	16.6	17.6	17.0	5.16	6.20	8.07	14.49	7.00
(n = 10†)	SD	1.4	1.6	1.8	1.7	0.69	1.32	2.28	5.63	0.86
1200	Mean	15.9	16.9	17.6	17.3	4.93	5.75	9.10	16.91	6.86
(n = 10)	SD	1.5	1.8	1.9	2.5	1.02	1.52	2.78	10.03	1.07
Test for Significance		NS	NS	NS	NS	NS	NS	NS	NS	NS

Abbreviations: DN, Duncan's multiple range test; NS, Not Significant; SD, standard deviation; SS, statistically significant compared to control.

†Week 4 Feed efficiency (n = 9)

*$p < 0.05$; **$p < 0.01$

Table S3. Organ Weights in the 28-Day Study

Group (mg/kg bw/day)		Body weight (g)	Organ weight (g)									
			Brain	Liver	Kidneys	Heart	Thymus	Spleen	Testes	Epididymides	Prostate†	Adrenals
**Male**												
Control (n = 10)	Mean	318.9	2.05	10.10	2.12	0.94	0.54	0.60	3.26	1.07	1.62	0.073
	SD	19.38	0.10	1.11	0.24	0.07	0.06	0.13	0.25	0.11	0.17	0.011
400 (n = 10)	Mean	325.1	2.01	9.91	2.05	0.94	0.54	0.62	3.18	1.07	1.69	0.076
	SD	16.72	0.05	1.07	0.16	0.08	0.10	0.07	0.28	0.14	0.32	0.013
800 (n = 10)	Mean	321.2	2.00	9.59	2.14	0.92	0.47	0.63	3.33	1.00	1.50	0.072
	SD	14.02	0.06	0.76	0.16	0.07	0.06	0.09	0.17	0.14	0.29	0.010
	SS						*					
1200 (n = 10)	Mean	324.9	2.01	9.95	2.16	0.93	0.48	0.63	3.36	1.09	1.61	0.078
	SD	20.65	0.08	1.16	0.18	0.10	0.07	0.06	0.20	0.13	0.25	0.007
Test for Significance		NS	NS	NS	NS	NS	DN	NS	NS	NS	NS	NS
Historical Control Range		249.0–316.0	1.90–2.21	7.09–9.92	1.69–2.55	0.70–0.98	0.49–0.93	0.49–0.77	2.84–3.54	0.66–1.07	0.86–1.39	0.050–0.087
**Female**									Ovaries		Uterus	
0 (Control) (n = 10)	Mean	202.8	1.91	6.03	1.51	0.70	0.42	0.47	0.100	–	0.61	0.087
	SD	12.24	0.09	0.52	0.14	0.06	0.07	0.05	0.016	–	0.16	0.008
400 (n = 10)	Mean	201.0	1.90	5.62	1.45	0.65	0.40	0.48	0.094	–	0.56	0.081
	SD	9.98	0.09	0.39	0.10	0.04	0.06	0.05	0.012	–	0.13	0.013
800 (n = 10)	Mean	201.3	1.90	5.85	1.43	0.66	0.44	0.43	0.090	–	0.56	0.081
	SD	15.87	0.08	0.76	0.12	0.05	0.10	0.05	0.020	–	0.16	0.010
1200 (n = 10)	Mean	203.8	1.87	6.02	1.41	0.63	0.41	0.49	0.095	–	0.52	0.082
	SD	20.35	0.09	0.86	0.13	0.06	0.09	0.10	0.016	–	0.15	0.012
	SS					*						
Test for Significance		NS	NS	NS	NS	DN	NS	NS	NS	–	NS	NS
Historical Control Range		160–195	1.75–1.98	4.74–6.61	1.20–1.61	0.54–0.66	0.28–0.59	0.32–0.54	0.081–0.113	–	0.35–0.56	0.055–0.099

Abbreviations: DN, Duncan's multiple range test; NS, Not Significant; SD, standard deviation; SS, statistically significant compared to control.

†Prostate with seminal vesicles and coagulating gland as a whole.

*$p < 0.05$

Remarks: Paired organs were weighed together.

Table S4. Organ Weights Relative to Body Weight in the 28-Day Study

Group (mg/kg bw/day)		Organ weight relative to body weight (%)									
		**Brain**	**Liver**	**Kidneys**	**Heart**	**Thymus**	**Spleen**	**Testes**	**Epididymides**	**Prostate†**	**Adrenals**
**Male**											
Control	Mean	0.646	3.162	0.663	0.295	0.169	0.187	1.020	0.337	0.508	0.023
(n = 10)	SD	0.047	0.229	0.054	0.012	0.021	0.035	0.039	0.027	0.035	0.003
400	Mean	0.618	3.044	0.630	0.290	0.167	0.192	0.978	0.327	0.520	0.023
(n = 10)	SD	0.023	0.231	0.041	0.012	0.030	0.020	0.087	0.038	0.103	0.003
800	Mean	0.623	2.982	0.666	0.286	0.145	0.195	1.038	0.313	0.466	0.022
(n = 10)	SD	0.034	0.121	0.032	0.017	0.016	0.023	0.059	0.049	0.081	0.003
	SS					*					
1200	Mean	0.621	3.057	0.667	0.286	0.148	0.193	1.039	0.337	0.495	0.024
(n = 10)	SD	0.053	0.212	0.057	0.026	0.023	0.017	0.104	0.050	0.067	0.002
Test for Significance		NS	NS	NS	NS	DN	NS	NS	NS	NS	NS
Historical Control Range		0.65–0.82	2.82–3.57	0.68–0.95	0.28–0.36	0.17–0.36	0.17–0.29	1.00–1.29	0.24–0.37	0.30–0.49	0.020–0.033
**Female**								**Ovaries**		**Uterus**	
0 (Control)	Mean	0.943	2.974	0.745	0.343	0.208	0.234	0.050	–	0.303	0.043
(n = 10)	SD	0.077	0.198	0.050	0.030	0.032	0.023	0.008	–	0.085	0.003
400	Mean	0.945	2.800	0.723	0.326	0.198	0.239	0.047	–	0.279	0.040
(n = 10)	SD	0.050	0.174	0.032	0.022	0.028	0.026	0.006	–	0.060	0.006
800	Mean	0.949	2.898	0.710	0.329	0.218	0.215	0.045	–	0.277	0.040
(n = 10)	SD	0.070	0.193	0.026	0.019	0.048	0.021	0.008	–	0.075	0.004
	SS			*							
1200	Mean	0.924	2.947	0.695	0.310	0.203	0.241	0.046	–	0.258	0.040
(n = 10)	SD	0.090	0.224	0.036	0.016	0.040	0.028	0.006	–	0.086	0.007
	SS			**	**						
Test for Significance		NS	NS	DN	DN	NS	NS	NS	–	NS	NS
Historical Control Range		0.91–1.19	2.83–3.72	0.66–0.89	0.28–0.37	0.15–0.34	0.19–0.32	0.043–0.059	–	0.19–0.31	0–0

Abbreviations: DN, Duncan's multiple range test; NS, Not Significant; SD, standard deviation; SS, statistically significant compared to control.
†Prostate with seminal vesicles and coagulating gland as a whole.
*$p < 0.05$; **$p < 0.01$

Table S5. Organ Weights Relative to Brain Weight in the 28-Day Study

Group (mg/kg bw/day)		Organ weight and body weight relative to brain weight (%)									
		Body weight	Liver	Kidneys	Heart	Thymus	Spleen	Testes	Epididymides	Prostate†	Adrenals
**Male**											
Control	Mean	15550.8	492.17	103.14	45.90	26.21	29.16	158.63	52.28	79.03	3.55
(n = 10)	SD	1089.95	54.54	10.94	3.75	3.06	6.46	12.30	4.96	8.68	0.51
400	Mean	16210.4	494.06	102.08	47.04	27.03	31.04	158.50	53.09	84.30	3.76
(n = 10)	SD	615.09	48.91	7.17	3.06	5.00	3.20	14.50	6.73	16.39	0.56
800	Mean	16089.4	480.45	107.18	46.08	23.33	31.39	166.74	50.04	75.00	3.62
(n = 10)	SD	866.33	42.19	7.61	3.73	2.81	3.89	8.87	6.05	14.39	0.56
1200	Mean	16203.5	496.50	107.70	46.25	23.81	31.27	167.22	54.04	80.23	3.89
(n = 10)	SD	1427.38	68.75	8.32	4.72	3.32	3.18	9.38	4.97	13.82	0.35
Test for Significance		NS	NS	NS	NS	NS	NS	NS	NS	NS	NS
Historical Control Range		12184–15340	344–492	83–133	34.3–48.2	24.5–47.7	23.8–39.1	140.2–171.8	33.8–51.9	41.0–69.5	2.4–4.4
**Female**								Ovaries		Uterus	
0 (Control)	Mean	10669.3	317.17	79.51	36.53	22.16	24.92	5.26	–	32.26	4.57
(n = 10)	SD	855.60	30.31	8.17	3.22	3.80	2.62	0.80	–	8.98	0.51
400	Mean	10613.5	297.62	76.66	34.55	20.98	25.31	4.96	–	29.61	4.28
(n = 10)	SD	559.36	30.08	4.54	3.14	2.82	2.72	0.78	–	6.48	0.72
800	Mean	10582.8	307.17	75.03	34.79	23.08	22.77	4.74	–	29.39	4.23
(n = 10)	SD	762.97	34.81	5.05	2.33	5.37	2.62	1.02	–	8.03	0.48
1200	Mean	10916.0	321.96	75.57	33.73	22.04	26.45	5.08	–	27.85	4.36
(n = 10)	SD	1089.27	42.85	5.53	2.97	4.41	5.49	0.94	–	8.44	0.55
Test for Significance		NS	NS	NS	NS	NS	NS	NS	–	NS	NS
Historical Control Range		8421–10971	249–378	65.9–92.0	27.3–37.7	15.1–33.0	17.3–30.5	4.24–6.46	–	18.3–29.5	2.88–5.38

Abbreviations: NS, Not Significant; SD, standard deviation.
†Prostate with seminal vesicles and coagulating gland as a whole.
Remarks: Paired organs were weighed together.
